# Report of Two Cases of Fracture

**Published:** 1887-11

**Authors:** F. M. Ridley

**Affiliations:** Lagrange, Ga.


### REPORT OF TWO CASES OF FRACTURE.

BY F. M. RIDLEY, M. D., LAGRANGE, GA.

In this day of conservatism, *asepsis*, the different resources and appliances of surgery, results are acquired in its practice which comparatively a few years ago would not have been hoped for. There is no part of surgery which requires greater care and attention to the accomplishment of successful results, and none which involves more serious results to the surgeon, as well as patient, than fractures. Dr. Samuel D. Gross, "the nestor of American surgery," said if called upon to testify under oath which branch of surgery he regarded as the most difficult to practice successfully and creditably, "he would unhesitatingly say that of the treatment of fractures."

In my humble experience no class of fractures has given me greater care and solicitude than those of the inferior extremity of the *humerus*. A simple fracture of this bone in this locality, with slightest injury to the soft parts, is sometimes attended with high inflammation, and the results of this morbid process, ultimate impairment of function, etc., etc.

The cases I report are laid before the profession:

1st. On account of its acknowledged unique results.

2nd. From the fact of its unusual occurrence and favorable treatment.

On the 2nd day of June last, I was called to visit Willie Morgan, son of Mr. John Morgan, a farmer living seven miles from this place. The patient was a youth twelve or thirteen years old, remarkably strong and healthy. About three hours before my arrival, he had been thrown from a mule into a pile of rocks, and the fall resulted in a compound fracture of the inferior extremity of the right *humerus*.

The bone was broken just above the condyles in a transverse direction, both ends of the fracture penetrating entirely the soft parts, and came out through the biceps in front, and the inferior fragment emerged through the triceps behind. The lad was suffering much pain; there was inconsiderable hemorrhage; the arm was much swollen and twisted. Circulation in the arm was good, and "the two great modifiers of repair, health and age," were in the youth's favor. I decided to endeavor to save the arm and amputate later if necessary. Ether was administered by my co-partner and brother, Dr. C. B. Ridley, to whose valuable assistance and opinion much of the success of the operation was due. After cleansing the parts thoroughly with a solution of bichloride of mercury and snipping off the jagged edges of the fracture, we coaptated the bones and applied a dressing of iodoform gauze, and otherwise observed the strictest antiseptic methods not necessary to detail here.

We applied the rectangular apparatus, and from day to day carefully watched the patient. About the 15th day we removed the dressings. Inflammation was high, of course, locally, and his temperature registered after first four days for six following from 101½ morning to 104 afternoon. For this elevation of temperature we gave the ordinary sedatives and frebrifuges and locally irrigated the parts with carbolized (20 per cent.) water. When the dressings were first removed there was some slight suppuration, which continued for several days. After the external wounds healed, we began to exercise passive motion of joint; this was continued from day to day. The lad was well nourished and nursed throughout, and now, about three months after the injury, it gives me pleasure to report him in perfect health with excellent motion of elbow-joint, and slightest possible deformity.

CASE NO. 2.—Albert Davis, a delicate lad of ten years, son of Mr. George C. Davis, who lives about four miles from this place, slipped from the back of a very tall horse, and while under the animal was either kicked or stepped upon.

I saw him about two hours after his fall, and found upon examination that he had sustained an oblique fracture of the upper third of the femur, which was complicated with dislocation backward of the head of this bone into ischiatic notch of Sir Astley Cooper.

With the efficient services of Dr. J. C. Johnson, a very accomplished young physician, and a recent honor-graduate of the Atlanta Medical College, I first reduced the dislocation by manipulation and rotation, after having patient thoroughly anæsthetized. I then coaptated the fracture, and applied a felt splint, and practiced extension by Buck's invaluable appliance, using sand-bags on either side of limb to steady it laterally.

After eight days I applied a bandage, not earlier on account of the inconvenience of the anticipated swelling, and this earliest possible time, because I find the bandage a material factor in the prevention of shortening. At the end of twenty-four days I removed the dressings and had the boy, with assistance, to move about the room on his crutches.

At this date, nearly four months after the accident, he has perfect use of his limb and not *the fractional portion of an inch* shortening by actual measurement.

